# History of medicine in medical education: new Italian pathways

**DOI:** 10.5195/jmla.2023.1586

**Published:** 2023-04-21

**Authors:** Silvia Iorio, Valentina Gazzaniga, Donatella Lippi

**Affiliations:** 1 silvia.iorio@uniroma1.it, Department Medico-Surgical Sciences and Biotechnologies, Unit of History of Medicine and Bioethics, Sapienza - University of Rome, Rome, Italy.; 2 valentina.gazzaniga@uniroma1.it, Department Medico-Surgical Sciences and Biotechnologies, Unit of History of Medicine and Bioethics, Sapienza - University of Rome, Rome, Italy.; 3 donatella.lippi@unifi.it, Department of Experimental and Clinical Medicine, University of Florence Studies, Florence, Italy.

**Keywords:** History of medicine, medical education, Italian case, medical humanities

## Abstract

**Objective:**

There is little doubt that there are currently obstacles in measuring the impact of the history of medicine within medical training. Consequently, there is a clear need to support a vision that can historicize Euro-Western medicine, leading to a greater understanding of how the medical world is a distinct form of reality for those who are about to immerse themselves in the study of medicine.

**Methods:**

History teaches that changes in medicine are due to the processes inherent to the interaction among individuals, institutions, and society rather than individual facts or individual authors.

**Results:**

Therefore, we cannot ignore the fact that the expertise and know-how developed during medical training are the final product of relationships and memories that have a historical life that is based social, economic, and political aspects.

**Conclusion:**

Moreover, these relationships and memories have undergone dynamic processes of selection and attribution of meaning, as well as individual and collective sharing, which have also been confronted with archetypes that are still able to influence clinical approaches and medical therapy today.

## OBJECTIVE

Today, despite the fact that nobody denies the usefulness of a philosophical or bioethical education for a future doctor, doubts have been expressed regarding the role that history can play in the co-construction of a new medical professionalism. Those who teach as humanists in medical departments are well aware of the fact that the main objection encountered in scientific circles to the usefulness of history in medical the training is linked to the hypothesis of a lack of impact of the humanities on experimentation and clinical practice.

In Italy, the main effects of this objection in the institutional field are the scarcity of funds, an inhomogeneous and rather limited space for the humanities in medical education, the shortage of teachers and sometimes their suboptimal preparation (due to the lack of official training for teachers in the medical-historical method, such as a doctorate).

Several international authors express alarming concern about the lack of quantitative evidence regarding the effectiveness of teaching Human Sciences in medicine and the related devaluation of the usefulness of an inclusive approach in courses. [1, 2, 3, 4, 5].

The same studies recognize the fact that it is nearly impossible to measure the impact and educational effectiveness of these sciences through tools currently adopted in medical education, identifying methodological obstacles that are likely insurmountable due to the vast plurality of possible misunderstanding. Consequently, there is a clear criticality in the measurement of the educational impact, which however does not exclude the need for greater clarity regarding the epistemological foundations, purposes, methods, and tools to be used in the training of students in the field of Human Sciences.

An interesting observation by Clayton J. Baker et al from 2017 proposes a complex evaluation approach, which not only uses an empirical, numerical, and taxonomic methodology, but also combines it with the collection of narratives on the individual experience of students of medicine [2].

These critical issues reflect a more general crisis of humanistic knowledge applied to medicine, also characterizing other cultural contexts in Europe and worldwide. For a historian, however, this situation is difficult only in appearance, being an important stimulus to reopen a discussion on the role of humanistic knowledge in medical training. Over time, history has responded to different needs and requirements of medical culture. Moreover, history has also provided the tools to respond to the strong need for professional legitimacy, giving doctors the means to respond to violent – not always unjustified – social attacks (i.e., the inability to “promise healing,” the ontological, conjectural, and procedural uncertainty of medicine before the Experimental Revolution, the construction and defense of the 'medical caste'). Last but not least, history has urged physicians to reflect on the desire to construct professional profiles aware of the importance of a constant update and reshaping in response to socio-cultural changing needs.

Classical Antiquity, history, and medicine have common origins and overlapping methodological pathways. Nourished by the same cultural climate and based on the same capacity for observation, medicine and history are based on a method of investigation that builds 'stories with meaning', linking the past with the present and the future. Therefore, it is essential to convey to medical students the idea that a historicist approach to problems in medicine has not only a value of cultural enrichment, but it is an indispensable tool to train experimental and clinical reasoning.

## METHODS

The history of medicine was born, in Italy, in the faculties of medicine. In Florence, in 1805, it appeared as “Philosophical history of medicine,” to educate on the exercise of logic and the “formation of a correct medical criterion” [6]; in Rome, A. Pazzini from 1932 began to hold optional courses of history of medicine addressed to medical students and founded in 1954 an Institute and a Museum.

The adjective “philosophical” that connotes this first phase of medical-historical studies in Italy, however, obscured by scientific nationalism, gives way over time to the easier way of antiquarian and celebratory recovery, transforming this border discipline into an anthology of “detached biographies, spicy anecdotes, edifying commemorations” (Lecourt 1972), that is, galleries of medallions. With time, the adjective 'philosophical' was lost and the History of Medicine became an antiquarian discipline, full of curiosities and anecdotes. While the 'precursor virus' claimed its victims, the history of medicine took the form of an 'amused inventory […] of the singularities of the past […]. It becomes an amiable and anecdotal erudition, to which the old doctor devotes his “well-deserved” free time, in which he relives the confused memories of the Latin lesson and the joys of college” (Starobinski 1953). This so-called history of medicine, common to the Historical Introductions of the scientific manuals, aimed at giving authority and prestige to the tradition, in the linear and simplistic way of a teleological vision.

Only following H.E. Sigerist and with a considerable delay compared to what happened in the United States and other European countries, since 1980 in Italy, the history of medicine has taken a path opening up to the work of professional historians [7]. While classical training disappears among doctors, a new historical-medical approach is built, rich in exegetical precision, critical interpretative potential, philological competence, and philosophical awareness of the method. At the same time, health historians, historians, and philosophers of science opened up new pathways that only partially cross those of the history of medicine taught in medical schools.

Today, there are different approaches, which can benefit from the contribution of other skills, basing their contributions on new needs in the framework of medicine, due to a status that has changed in a noteworthy manner. Scientific knowledge – precise, exact, ready to replace old theories with newer ones – has been measured against other needs and illnesses, beyond the biological norm. At the same time, the history of medicine has begun to consider the anthropological and social implications on which it has learned to renew its methods, contents, and objectives. Therefore, there emerges the need to recognize a vision that can historicize the Euro-Western medical art, that is, trying to understand how the medical world comes to be composed as a distinct form of reality for those who are preparing to immerse themselves in the study of medicine [8]. Moreover, the history of medicine has learned to relocate itself within the broader framework of the humanities, which are useful for the training of doctors, in order to learn the tools of an interdisciplinary conversation and adapt its research methods to transdisciplinary issues. Within this context, the rather sterile debate on the cultural characteristics of those who intend to research and teach in the medical-historical field no longer makes sense. The discipline is open to anyone who possesses the methodological requisites required by the historical sciences and by philology, understood as the ability to study the understood or misunderstood meanings of phenomena, ideas and relations between individuals and society.

### History of Medicine and MH

The history of medicine has today found a solid place in the broader field of MH. In its new guise, it teaches how to listen and narrate, to understand (verstehen), and to explain (erklären). The history of medicine shows that the concept of “evidence” does not only refer to empirical and statistical evidence but also to immediate judgement and it is valid for diagnosis, therapy, the patient-doctor relationship, and research. The historical-narrative paradigm of the Medical Humanities is now widely recognized as a necessary and complementary tool to the technical dimension of the medical act. If medicine is a “specialist culture” in which communicative and relational data play a fundamental role, it is possible to use technical and instrumental data in an optimal manner, building scientific evidence in relation to the history of the individual patient.

From a medical perspective aimed at addressing the overall treatment of the disease, a renewed history of medicine, reallocated within the broader field of Medical Humanities, becomes a transversal and privileged tool to understand and transform the patient's history in a true medical history, re-evaluating the narrative competence against the exclusive dominance of the logical-scientific ability. History can contribute to train the doctor in the use of tools or 'semiological codes' that go beyond the spoken word and the learning of the principles and application of counseling, overcoming the tendency of medical language to 'objectify' the patient and overestimate the dimension of disease when compared to that of the illness [9].

It is a question of framing the history of medicine in its dimension of 'critical consciousness' of the doctor's training. We have just lived through the experience of a pandemic in which history was often invoked in an attempt to clarify whether the study of past material could be used as a predictive model. Historians of medicine today generally share the position taken many years ago by Mirko D. Grmeck [10]: medicine needs a history understood as a tool for understanding what, in a more or less conscious way, remains of the past in the structures, ways of thinking, conceptualisations of today. We need a 'contemporary' history, that is, history that is able to pose questions that make sense only if they can decode the constantly changing actuality that requires to be questioned in ways always renewed. If we accept this point of view, the pandemic has taught us what Walter Benjamin wrote in 1940: Paul Klee's painting entitled Angelus Novus shows an angel who seems to be in the act of moving away from something he is staring at. His eyes and mouth are wide open, his wings are outstretched. The angel in the story must look like this. His face is turned towards the past.

## RESULTS

In order to answer this question, we have some tools at our disposal. For example, the possibility of exploiting the results of a historiographic debate dating to the twentieth century on the meaning and usefulness of history. It is a discussion involving the definition and the purposes of historical investigation. Is history a search for objective truths set in time or an attempt to subtract facts from the subjectivity of the historian's own account, which always suffers from prospective visions? Is it a partial and fallible knowledge whose value lies precisely in its not being enduring over time and always ready to change? Is history a tool used in order to avoid repeating mistakes, or is it the key to understanding how the present reveals its connections with the past? Or is it the tool to read the mistake as an opportunity for a turning point, in order to understand that oblivion is one of the forms of memory [11]. Or lastly, should more attention be paid to the study of fractures and lack of continuity rather than focusing on accumulated successes and continuities of facts and thoughts?

Each of these questions is also useful if applied to the history of medicine. They can obtain valid answers to explain why a methodologically correct historicist approach is not only useful but also indispensable for the doctor in training. A well-documented reference to the pedagogical necessity of a new, more solid history of medicine is in the thorough examination carried out by Jones et al. in 2014 in the Journal of the History of Medicine and Allied Science [12]: beyond the clichés proposed by historians of medicine, the authors insist on the potential that history has to explain the social character of medical science and it being a product and producer of processes of a cultural, political, and economic nature. The secular and constant dialogue of medicine with its socio-cultural contexts has produced an interaction from which the concepts of gender, race, and class have emerged. Moreover, medicine has contributed to the structuring of the debate on gender, sex, sexuality, and its control, while also defining the role of doctors and determining the ways in which they have conceived and organised places of care and health systems. Medicine has created diverse and dynamic healthcare markets, collaborating with other specialist cultures in defining concepts and meanings of illness, which change over time, shaping social individuals and conditioning their responses. Medicine has contributed to constructing and deconstructing images of the bodily self, as the same time shaping patientsbehaviors and influencing their demands for health. Lastly, it has influenced public perceptions of issues relating to treatment and healing and affected societies' expectations of doctors' technical skills.

In short, a young person cannot ignore the fact that the area of expertise in which he or she is being trained is the end product of relationships and memories that have a “historical life,” which have been subjected to dynamic processes of selection and attribution of meaning and to individual and collective sharing, and which have been confronted with archetypes that can still influence clinical and therapeutic practice. History teaches that changes in medicine are due to processes of the interaction among individuals, institutions, and society rather than changes caused by single facts or single authors. As for any other historical fact, the change occurs by the abandonment of long-term ideas and practices, which generates paradigm shifts and triggers complicated processes in which preservation of cultural identity and oblivion of some of its aspects and moments are mixed in a dynamic and unstoppable process.

### What History Means for a Doctor

History offers a considerable advantage to the training and formation of a doctor. Proper training creates the foundation for clinical reasoning. It has been said that medicine and history have common origins and characteristics: Croce said that they are the only disciplines in which the researcher subject coincides with the object of study. The idea that history is a “science *sui generis*,” based on facts, their representation, and logical connection, is also applicable to the intellectual domains of medicine: for historians, facts are erudition and documents, representation is their interpretation and narration, and their random connection generates history [13]. In a clinical investigation, these three dimensions correspond respectively to semeiotics, symptomatology, the attention to the psychological dimension that determines the experience of illness, and lastly diagnosis. For the historian, the search for truth is a dynamic process made up of observation, interpretation, and transmission. However, this pursuit is also defined by the choice of what can be overlooked or forgotten, which nevertheless continues to construct individuals and cultural communities [14]. This process is simple only in appearance, because in fact it is the “fruit of different mental operations” [15]; for this reason, it is not dissimilar to the method of problem solving based on the distance from the “common sense that is generated by mere experience” on “organic cognitive systems” that produce specialized knowledge capable of combining their expertise to answer given questions (what in literature is indicated as “powerful knowledge”).

In short, if history is seen as a method capable of correctly asking questions with a sense of current events, not necessarily in possession of neutral and objective sources on which to base one's ideas and give certain answers, history becomes a formidable training tool for clinical reasoning. In history, medical students can find a conjectural and provisional model of knowledge that can overlap the methods and means of experimental reasoning and repercussions in clinical reasoning. As clinicians know, ”…the meaning, besides referring to real things such as symptoms, extends to other 'types of objects', that is to other realities, such as sensations, impressions, emotions, considered as real as symptoms, even if they belong not to those who are ill but to those who care. From a logical point of view, these 'types of objects' cannot be distinguished from the others, those that are symptomatic. They all contribute to the development of meaning. From the clinical meaning, it is not easy to exclude the intentional component of the doctor. This is due to the fact that it is impossible, in the name of an absolute objectivity of the symptom, to exclude what the doctor intends to say by attributing its meaning to it.” [16]

If the goal in medicine is the push towards objectivity, this is often a deceptive mirage. Like the historian, the clinician does not ask objective questions and the sources on which his analysis is based are the result of a selection between signifier and non-signifier, sign and symbol, biological datum, and psychological datum. They are the result of a selective process that is the orientation of how one sees the data that appear to be more useful in directing one's research towards the truth. The historian, much like a doctor, carries out their work within an intellectual realm without absolute truths, objectivity, and stability of knowledge – in history and medicine, what we see is always quite loaded with theory. The experimental method, like that of history, deconstructs myths and dogmas, trains people how to doubt, and is an “organized practice of dissent,” as Bloch said [14]. Historians and physicians use the same method to create stories that reconnect the cultural or biological past with the problems of the present. The perception of the present is in both cases the first step for prognostic processes, or in other words, predictive processes.

The idea of a dynamic method, based on facts that are constructs that require constant reassessment, brings together some positions of twentieth-century historiography [17] with the epistemology of T. Kuhn. The common trait is a method that “does give certainty to that which what is doubtful and does not generalize isolated cases” defines a field of knowledge that, like medicine, is precisely historical, meaning that it is evolving, incomplete, fallible, and falsifiable [17]. The documents on which historical knowledge is founded are as silent as the signs and symptoms for A. Murri (if the historian or physician cannot ask the correct questions) [6]. The case (the same that in the history of medicine reveals, for example, I. Semmelweiss the secret of puerperal fevers) appears to be the dimension to which the historian must be opened in order to accept unexpected documents, capable of pushing the reformulation of the initial hypotheses and the evaluation of new explanatory models through experimental and factitious elements [18].

These comparisons allow for the usefulness of history to emerge as a training ground for medical education in the critical sense. The means not believing what appears to be true at first sight, seeking “the secret connections that bind things,” verifying hypotheses by dismembering facts, moving forward in the analysis of sources from the most superficial level of their reading to the more structured and deeper levels. C. Ginzburg wrote beautifully on the cognitive process that associates medicine with philosophy, jurisprudence, iconology and iconography and the doctor to Sherlock Holmes and the art connoisseur [18]. Consequently, the search for truth passes, in very different domains, through the common ability to select specific things, attributing meaning to what is apparently non-significant or even to what is absent, in order to search for the silent signals (symbols, images, or signals from the body) that shed light on reality. The result of this process is a circumstantial paradigm, made up of questioning, consultation of witnesses, formulation of hypotheses, their setting aside and their replacement. Therefore, reliable knowledge is produced because it is democratic, that is, open to the control of the cultural communities, which are the only ones competent to validate the survey results, for the time in which those results are acceptable.

Therefore, working within a historical domain allows the medical student to place the profession in the right perspective of the relativism of knowledge, as well as the perception of the epistemological uncertainty that continues to characterise medicine, despite its progressive “scientification,” of the temporal expiry of knowledge. History teaches that nothing of what is observed and seen has a univocal value, that some forms of the past are useful for explaining the present, while others have lost their meaning and can be ignored. The correct application of the historical method makes it clear that the constant reformulation of the concepts of health and disease depends on biological-scientific evidence, but also on cultural and social meanings: that which is illness in the past today is no longer so, or a disease or illness in a culture may not be so in another [19; 20; 21;22]. Along these lines, an illness or disability may also depend on the social and organisational decisions and strategies taken or not taken to counteract their effects.

Last, but certainly not least, the understanding and study of everything that has made medicine, when viewed over a long period of time, a complex field of study can provide the student with the tools to avoid the risk of the “presentisation” of our time, that is, the habit of trying to solve problems before understanding them [11]. Mankind and time, the subject of study in medicine and history, tenaciously refuse to be reduced to the concepts of norm, regularity, and simplification, on which a large part of scientific medicine rests. An eccentric voice – which leads to the indefinite verification of all the evidence, reminding doctors of the provisional nature of their knowledge through the representation of everything extraneous, even in the recent past – can only help in the construction of applicative models and renewed forms of experimental reasoning.

## CONCLUSIONS

Therefore, history can be seen as an indispensable tool for questioning modern medicine. However, studying certain aspects as they are represented by their own past, using a method that exploits the pedagogical potential provided by errors, divisions, as well as changes and interruptions of continuity in Western medical thought, might represent a greater challenge than initially expected.

Historical approaches and discourses have been weakened by a general crisis that has led to its widespread delegitimization as apparently unable to provide explanations pertinent to today and above all unable to avoid the repetition of errors and horrors with which humanity is already confronted. Moreover, the historical discourse suffers from the conditions that define our times, in which ephemeral news accumulates, destined – as seen on television or virtual platforms – to be rapidly supplanted by other news and other information that are equally volatile.

Therefore, historical discourse must find new ways of working in a world that – especially in the expectations of first year medical students - appears entirely foreign and, at times, openly hostile: “I thought I no longer had to study history” or “I have started studying medicine because the humanities don't interest me” are comments that are fairly well known to those of us who work as historians with medical students. This actually a resistance, as well as a challenge, that can be circumvented, provided that we have learnt to use some additional tools. The solution often begins with the need to deconstruct the introjected image that students have of the humanities.

As a result, what are the means at our disposal to push medical students towards 'thinking historically', as Brusa [15] suggests first and foremost, we need to change our methods and ask ourselves new questions. Beyond the scientific interests that characterise our research, we also need to convert our knowledge in order to create a dialogue with basic and clinical disciplines. Moreover, we need to try to grasp what are the possible points of intersection and topicality on which to work in order to convey historical contents that have any real use for medical students. We must be able to see that the youthful attitude of having our feet immersed in an immediate and eternal present may not be entirely damaging. On the contrary, this attitude can be used in our favour, reformulating questions that regard aspects that are somehow significant for our audience. Literature suggests that these questions do not necessarily have to be 'medical', but rather be based on themes of social interest that are potentially attractive to a young audience. Where does violence against women and the concept of female inferiority come from? What is technical language, and how did it arise? And are its goals or aims in solving problems? What were the lifestyles of the past and how did they affect health conditions? How is the image of the body built culturally? And how is a category of marginality built? Can culture be the cause or product of disease/illness? Do diseases or illnesses have a history? What sources are available to us to try to reconstruct this biological and cultural history? How are inequalities generated historically? Do political facts and social events affect health care choices? Do the relationships between human and ecosystems have effects on health? What are the relationships between time and medicine? What relationships do the concepts of miasma, contagion, guilt and sin have?

Each of these questions could potentially generate a monographic course during a semester, and each represents an opportunity to chip away at the ‘youthful idea' that the past is made up of a dusty accumulation of data to be learned - somehow 'memorable' names, book titles, years and events. Each question also has the potential to illustrate how even the most recent past is a foreign land from which we are irreparably separated [23]; and each question has the potential to show how – quite to the contrary, and in a hidden manner, often something we are unaware of – that past is still something that we take in and breathe, feeding thoughts and generating preconceptions even today.

Moreover, each question has the potential to illustrate the true historical path of medicine, made up of changes, fractures, chasms, and steps backward, the sense of the connection between facts and ideas, and the modification of the concept of causality. Consequently, these questions can correct the mythical image of medicine that advances with “magnificent and progressive fortunes.” The positivistic, often naive, attitude of the students cannot be ignored, as well as the 'presentism' of generations raised in virtual worlds that are always accessible, where everything seems close at hand and everything seems real, and where – as Prosperi reminds us – each level of competence seems the same as the other, and the other is superimposable [11]. The inability to distinguish between true and false, to verify the reliability of a source, to distinguish between signifier and non-signifier, are all issues to be addressed seriously and in depth, if we are to properly train those who choose to become a doctor – or perhaps, more trivially, those who work as doctors.

Therefore, the real pedagogical issue is to convince students heading down the scientific path that anyone who lives in the present “is making history and lives in a universe of historical processes,” as well as that the history of medicine, much like clinical studies, is a tool available for construction of identity and which cannot remain extraneous. The knowledge and understanding of the processes through which medicine is made up – as a form of specialised culture and as the result of the conceptualisations and actions of a social group capable of informing the surrounding world of itself, generating theories and consequent behaviour – creates an intellectual framework that helps the tomorrow's doctors see themselves within a shared identity [24, 25, 26, 27]. This takes place at the same time as increasingly sophistication specialisations are building even more fragmented and seemingly self-sufficient identities.

The possible and expected outcomes and goals of this attempt redefine historical-medical teaching are the following: an increasingly critical awareness of medical 'sources'; the ability to confidently move within the enormous pool of scientific data available constantly increasing and which must be 'diagnosed', in the ancient sense of the term; the increase of knowledge and understanding of the conscious use of medical terminology, in order to convey clear and understandable meanings, which respond to the real information and psychological needs of patients. This also means that a doctor must also understand their fears and the possible overload of implicit meanings that are inherent to disease and illness. Lastly, the history of medicine taught to future doctors is aimed at the acquisition of a sense and understanding of the complexity of medical actions, soliciting attentive responses to the scenarios and situations of care and protecting physicians from the risks that are constantly lurking along the blurry lines that separate health, life, disease, illness, and death.

## References

[1] Batistatou, A., Doulis, E.A., Tiniakos, D., Anogiannaki, A., Charalabopoulos: “The introduction of medical humanities in the undergraduate curriculum of Greek medical schools: challenge and necessity.” Hippokratia, no 14(4), pp. 241–243 (2012). PMC3031316 21311630

[2] Baker, C.J., Shaw, M.H., Mooney, C.J, et al. “The Medical Humanities Effect: a Pilot Study of Pre-Health. Professions Students at the University of Rochester.” J Med Humanit, no 38, pp. 445 (2017). 28589308 10.1007/s10912-017-9446-4

[3] Belling, C.: “Commentary: Sharper Instruments: On Defending the Humanities in Undergraduate Medical Education.” Academic Medicine, no 85(6), pp. 938–940 (2010). 20505388 10.1097/ACM.0b013e3181dc1820

[4] Bleakley, A.: “When I Say…the Medical Humanities in Medical Education.” Medical Education, no 49(10), pp. 959–960 (2015). 26383067 10.1111/medu.12769

[5] Charon, R.: “Commentary: Calculating the Contributions of Humanities to Medical Practice-motives, Methods, and Metrics. Academic Medicine.” Journal of the Association of American Medical Colleges, no 85(6), pp. 935–937 (2010). 10.1097/ACM.0b013e3181dc1ead 20505387

[6] Murri, A.: Scritti medici. Gamberini e Parmeggiani Editore, Bologna (1902).

[7] Caramiciu, J., Arcella, D., Desai, M.S.: “History of medicine in US medical school curricula.” J Anesth Hist, no 1(4), pp. 111 (2015). 26828087 10.1016/j.janh.2015.02.010

[8] Ousager, J., Johannessen, H.: “Humanities in Undergraduate Medical Education: A Literature Review.” Academic Medicine, no 70, vol. 6, pp. 988–998 (2010). 10.1097/ACM.0b013e3181dd226b 20505399

[9] Bates, V.: “Yesterday's Doctors: The Human Aspects of Medical Education in Britain,” 1957–93. Med Hist, no 61(1) (2017). 10.1017/mdh.2016.100 PMC5206963 27998331

[10] Grmeck, M.: La vita, la malattia e la storia. Di Renzo Editore (1998).

[11] Prosperi, A.: Un tempo senza storia. Torino, Einaudi (2021).

[12] Jones, D.S., Greene, A., Duffin, J., Warner, J.H.: “Making the Case for History in Medical Education.” Journal of the History of Medicine and Allied Sciences, no 70, vol 4, pp. 623–652 (2014). 25395574 10.1093/jhmas/jru026

[13] Jones, D.S, Greene, J.A., Duffin, J., Warner, J.H.: “Making the case for history in medical education.” JHist Med Allied Sci, no 70(4), pp. 623–652 (2014). 25395574 10.1093/jhmas/jru026

[14] Bloch, M.: Cosa chiedere alla storia. Roma, Castelvecchi (2016).

[15] Brusa, A.: “L'alfabetizzazione storica. Leggere storia e pensare storicamente.” In: “Utilità e inutilità della storia.” Il Bollettino di Clio, no 15 (giugno), pp. 52. (2021)

[16] Cavicchi, I: Filosofia della pratica medica. Milano, Bollati Boringhieri (2002).

[17] Lauria, P.: “La verità, tutta la verità, nient'altro che la verità,” Arnaldo Momigliano, Carlo Ginzburg e il compito dello storico (2014), www.giornaledistoria.net. last accessed March 4 2022

[18] Ginzburg, C.: Miti, emblemi e spie. Morfologia e storia. Torino, Einaudi (2000).

[19] Ackerknecht, E.H.: “The role of Medical History in Medical Education,” Bulletin of the History of Medicine, no 70, vol 2, pp. 135–145 (1947). 20242548

[20] Albury, W.R.: “Broadening the Vision of the History of Medicine,” Health and History, no 7(1), pp. 2–16 (2005).

[21] Barsu, C.: “History of Medicine between tradition and modernity.” Clujul Medical, no 90(2), pp. 243–245 (2017). 28559713 10.15386/cjmed-794 PMC5433581

[22] Beccalossi, C., Cryle, P.: “Recent Developments in the Intellectual History of Medicine: A Special Issue of the Journal of History of Medicine.” Journal of the History of Medicine and Allied Sciences, no 67(1), pp. 1–6 (2012). 21724642 10.1093/jhmas/jrr020

[23] Lowenthal, D.: The Past is a Foreign Country. Cambridge, Cambridge University Press (2015).

[24] Ludmerer, K.M.: “The History of Medicine in Medical Education.” J Hist Med Allied Sci., no 70, pp. 656–660 (2015). 26363047 10.1093/jhmas/jrv035

[25] Orefice, C. Banos, J.E.: The role of the humanities in the teaching of medical students, Dr. Antoni Esteve Foundation, Mon. 38, Barcelona (2018).

[26] Shedlock, J., Sims, R.H., Kubilus R.K.: Promoting and teaching the history of medicine in a medical curriculum. JMed Lib Assoc., vol. 100, pp. 138–141 (2012). 10.3163/1536-5050.99.100.2.014 PMC3324806 22514512

[27] Von Engelhardt, D.: “Teaching History of Medicine in the Perspective of “Medical Humanities.” Croatian Medical Journal, no 40(1) (1999). 9933888

